# A randomized controlled trial evaluating the effects of transversus abdominis plane block with compound lidocaine hydrochloride injection on postoperative pain and opioid consumption and gastrointestinal motility in patients undergoing gynecological laparotomy

**DOI:** 10.3389/fnmol.2023.967917

**Published:** 2023-01-25

**Authors:** Linlin Zhang, Zhen Jia, Tianyu Gao, Yigang Wang, Yuying Zhao, Jing Li, Yonghao Yu, Qing Li, Guolin Wang

**Affiliations:** ^1^Department of Anesthesiology, Tianjin Medical University General Hospital, Tianjin, China; ^2^Tianjin Research Institute of Anesthesiology, Tianjin, China

**Keywords:** compound lidocaine injection, TAP block, postoperative pain, nausea and vomiting, opioid consumption

## Abstract

**Introduction:**

Incorporation of transversus abdominis plane (TAP) block into multimodal analgesia has been emphasized in Enhanced Recovery protocols (ERPs). However, benefit is limited in clinical practice. A potential explanation is the short duration of analgesia of standard local anesthetics. Herein, this randomized, double-blind, controlled trial evaluated whether TAPB with long-acting compound lidocaine hydrochloride injection reduces postoperative pain.

**Methods:**

164 patients undergoing elective gynecological laparotomy under sevoflurane anesthesia randomly received ultrasound-guided TAP block with either saline, or ropivacaine, or compound lidocaine before anesthesia induction. The postoperative pain intensity (primary outcome) was evaluated by pain 11-point numerical rating scale. We also recorded sufentanil consumptions, time to first flatus, side-effects and hospital stay after surgery.

**Results:**

We reported that pain scores at rest at postoperative 3h in group 0.375% ropivacaine was lower than that in group saline [mean 2.4 (SD 1.2) *vs.* 3.0 (1.0), *p* = 0.036]. Compared with saline, 0.4% and 0.6% compound lidocaine caused lower pain scores at rest at postoperative 12h [2.8 (0.9) *vs.* 2.1 (0.9) and 2.0 (0.9), *p* = 0.016 and *p* = 0.006]. Sufentanil usage for the first postoperative 48h was lower in group 0.6% compound lidocaine than group saline [24.2 (5.4) *vs.* 45.6 (7.5) µg, *p*  < 0.001]. Time to first flatus and hospital stay after surgery was shortest and the incidence of postoperative nausea was lowest in patients receiving 0.6% compound lidocaine.

**Conclusion:**

TAP block with 0.6% compound lidocaine hydrochloride injection attenuates postoperative pain, reduces opioid consumption, accelerates gastrointestinal function recovery, and shortens length of hospital stay in patients after gynecological laparotomy.

**Trial registration:**

ClinicalTrials.gov, identifier: NCT04938882.

## Introduction

Acute pain after surgery is practically omnipresent. According to statistics, more than 40% of patients undergoing surgery experience moderate or severe pain in the immediate postoperative period (Small and Laycock, 2020). Poor postoperative pain control is a leading factor that hinders physiological recovery and causes chronic postsurgical pain, which its prevalence is nearly 10% after all surgeries (Glare et al., 2019). Chronic postsurgical pain not only causes the discomfort and disability of patients, but also represents a vast potential for psychological, social and economic adversity (Sinatra, 2010). Thus, an essential goal is to provide adequate analgesia after surgery.

Opioids are the current mainstay for perioperative analgesia but have several side effects and can predispose patients to problematic long-term use (Colvin et al., 2019; Kent et al., 2019). Enhanced recovery protocols (ERPs) using evidence-based surgical principles have been indicated to decrease postoperative morbidity and length of hospital stay (Beverly et al., 2017). Accumulating evidence emphasizes that multimodal analgesic strategy, as a critical component of ERPs, is required for earlier functional recovery through reducing opioid-related adverse effects (Wu et al., 2019; Allen et al., 2020). However, it will be of great importance that these benefits from minimizing opioid consumption should not come at a cost of impairing optimal analgesia after major surgery.

Transversus abdominis plane (TAP) block is a regional anesthesia technique that provide analgesia of the parietal peritoneum, the anterior abdominal wall and the skin (Tran et al., 2019). Recently, incorporation of TAP block into multimodal analgesia has been touted as promising therapeutics in patients with postoperative pain after open or laparoscopic abdominal surgery (Oksar et al., 2016; Edney et al., 2019). However, benefit is limited in clinical practice. An underlying explanation is the restricted duration of analgesia of standard local anesthetics, such as ropivacaine and bupivacaine (Golembiewski and Dasta, 2015).

Menthol is well recognized for its pharmacological properties of persistent analgesia and anesthesia *via* cumulative inactivation of voltage-gated sodium channels, blockade of neuronal calcium ion channels, inhibition of inflammation, and desensitization of nociceptive receptors (Gaudioso et al., 2012; Liu et al., 2013; Oz et al., 2017; Pergolizzi Jr. et al., 2018; Wright et al., 2018). Furthermore, menthol has become the primary active ingredient and permeation enhancer for several topical analgesic formulations in clinics (Castro and Dent, 2017; Pergolizzi Jr. et al., 2018). Compound lidocaine hydrochloride injection is a long-lasting formulation containing lidocaine, menthol and glycerin, which has been approved by the Chinese Food and Drug Administration (Drug Approval Number: H10940072) for intraoperative anesthesia and postsurgical analgesia in adults through local wound infiltration (Wang, 2015, 2020; Dai et al., 2019). However, TAP block with compound lidocaine has never been investigated for the alleviation of postoperative pain in the clinical setting. We hypothesized that compound lidocaine would reduce pain intensity and opioid use in patients undergoing gynecological laparotomy. Our findings may offer a possibility for a novel recommendation for postoperative pain control in patients after abdominal surgery.

## Materials and methods

### Study design

This study was approved by the Tianjin Medical University General Hospital Ethic Committee (Tianjin, China; Approval Number: IRB2021-YX-094-01), and the study protocol was registered^1^ (Identifier: NCT04938882), and written informed consent was obtained from all patients.

Patients were randomly allocated to one of four groups: patients in Group S receiving TAP block with saline (0.9% sodium chloride injection); patients in Group 0.375% R receiving TAP block with 0.375% ropivacaine; patients in Group 0.4% CL receiving TAP block with 0.4% compound lidocaine; patients in Group 0.6% CL receiving TAP block with 0.6% compound lidocaine. A bilateral, classic TAP block was performed with a volume of 40 mL reagents (20 mL for each side) under direct ultrasound (US) visualization before the induction of anesthesia. As the manufacturers’ instructions, 0.75% ropivacaine hydrochloride injection (10 mL; Jiabo Pharmaceuticals Co., Guangdong, China) contains ropivacaine 75 mg; 0.8% compound lidocaine hydrochloride injection (10 mL; Tianjin Jinyao Pharmaceuticals Co., Tianjin, China) contains lidocaine 80 mg and menthol 13 mg.

All patient assignments were guided by a computer-generated random number system and individually sealed envelope. Study medication were provided by the hospital pharmacy. TAP block was administered by an experienced anesthesiologist not involved in the intraoperative management and data collection. Patients were blinded to the group allocation. Primary and secondary outcomes were assessed by another anesthesiologists responsible for the data collection, but not directly involved in the treatment of the patients and who was blinded to randomization.

### Study inclusion and exclusion criteria

Patients undergoing elective exploratory laparotomy for gynecological cancer were screened and enrolled between August 23, 2021 and February 10, 2022. Inclusion criteria were patients aged 20–60 years; American Society of Anesthesiologists physical status I or II; cognitive capacity to use the patient-controlled analgesia (PCA). The exclusion criteria were as follows: bronchial asthma; coronary heart disease; severe hypertension; diabetes mellitus; obesity (BMI >30 kg/m^2^); cardiac, hepatic, and renal dysfunction; psychiatric disease; history of chronic pain; history of alcohol or opioid abuse; chronic use of opioids; intake of any analgesic within 48 h before surgery; pregnancy; allergy and contraindication to any drug used in the study; local infection at the TAP block site; contraindication for the use of PCA; or incapacity to comprehend pain assessment. After randomization and allocation, patients were withdrawn if re-detection for postoperative bleeding or if protocol was violation.

### Interventions and anesthesia

All surgical procedures were performed by senior surgeons. Patients fasted preoperatively. Acetaminophen 500 mg was given orally in the preoperative holding area. Upon arrival at the operating room, the patients were generally monitored by non-invasive blood pressure, ECG, heart rate (HR), pulse oximetry, and bispectral index (BIS). A peripheral intravenous line in the non-dominant arm were attached before anesthesia induction and urinary catheter were placed after induction of anesthesia. All patients received a bilateral US-guided TAP block. After skin disinfection and preparation of sterile field, A 21-gauge, 110-mm, sonographic needle was then inserted in plane and directed toward the fascia between the transversus abdominis and the internal oblique muscles; drug injection was performed at each side under direct US visualization.

Induction was performed with midazolam 0.05 mg·kg^−1^, sufentanil 0.2 μg·kg^−1^, and propofol 2.0 mg·kg^−1^, and tracheal intubation was facilitated with rocuronium 0.7 mg·kg^−1^. After intubation, all the patients were mechanically ventilated [end-tidal carbon dioxide values of 35–45 mmHg]. Flurbiprofen axetil (50 mg), one of nonsteroidal anti-inflammatory drugs (NSAIDs), was intravenously administered before incision. A continuous infusion of remifentanil (0.1 μg·kg^−1^·min^−1^) was maintained for intraoperative analgesia, allowing rate adjustments (±0.05 μg·kg^−1^·min^−1^) based on targeting hemodynamic changes: HR exceeding pre-induction values by 15% and mean arterial blood pressure (MAP) exceeding baseline values by 20% for at least 1 min. Anesthesia was maintained with sevoflurane (Maruishi Pharmaceutical Co., Osaka, Japan) as an initial 1.3 minimal alveolar concentration (MAC) and oxygen–air mixture (fraction of oxygen, 50%). The depth of anesthesia was adjusted during surgery by 1% stepwise titration of sevoflurane, based on targeting BIS (45–60). Intravenous rocuronium (0.3 mg·kg^−1^) was administered intermittently during anesthesia. Intraoperative fluids (2–4 mL·kg^−1^·h^−1^) were administered *via* infusion pumps with a goal of zero fluid balance. If bradycardia (HR <45 beats·min^−1^) and continuous hypotension (MAP <60 mmHg) persisted, additional fluid infusion, atropine (0.5 mg), and phenylephrine (0.1 mg) were also administered. During skin closure, sevoflurane and remifentanil were stopped, and tropisetron (2 mg) was injected for the prophylaxis of postoperative nausea and vomiting (PONV). Residual neuromuscular block was antagonized by neostigmine 0.04 mg·kg^−1^ and atropine 0.01 mg·kg^−1^ when the tidal volume of spontaneous breathing exceeded 200 mL. When BIS value reached 80, response to oral command was observed, followed by eye opening and spontaneous breathing rate exceeding 10 breaths·min^−1^, the patient was extubated and moved to the postanesthetic care unit (PACU) for recovery at least 1 h.

### Outcomes

The primary outcome was pain intensity at the different time intervals after surgery. Pain intensity was evaluated on an 11-point numerical rating scale (NRS): 0 = no pain; and 10 = worst pain imaginable (Zhang et al., 2016). The NRS score for pain at rest and after movement was assessed at 1, 3, 6, 12, 24, 48, and 72 h after surgery. Movement was specified as active mobilization and weight bearing while escaping any harm (Zhang et al., 2016). First postoperative pain (NRS > 4) in the PACU was primarily managed by sufentanil titration, which was administered in 3 μg doses at intervals of 3 min until NRS < 4 (Zhang et al., 2016, 2017). However, sufentanil titration was discontinued if the Ramsay score (1 = anxious and agitated or restless, or both; 2 = cooperative, oriented, and tranquil; 3 = responds to command only; 4 = asleep, but has a brisk response to light tactile stimulus or a simple verbal command; 5 = asleep, but arousable only by strong physical stimulus; and 6 = asleep, unarousable) was >3, peripheral oxygen saturation decreased <92%, or breathing rate was <10 breaths min^−1^ (Zhang et al., 2016, 2017). Total dose of first postoperative sufentanil in the PACU were documented. All patients received scheduled intravenous flurbiprofen axetil (50 mg) every 12 h for 72 h following surgery. Oral acetaminophen 500 mg was also administered every 8 h starting from postoperative 6 h. Furthermore, each patient was administered analgesics using a PCA pump containing sufentanil (200 μg) diluted by saline to a total volume of 200 mL after discharge from the PACU for 72 h. The device was set to deliver a basal infusion of 2 mL h^−1^ during the first 3 h, and bolus doses of 0.5 mL with a 15 min lockout period was given as needed for 72 h. Intravenous sufentanil (5–10 μg) as rescue analgesia was available as needed for breakthrough pain. Total sufentanil comsumption was recorded through 6, 12, 24, 48, and 72 h after surgery.

Time until passage of flatus, time until urinary catheter removal, time to mobilization, and the incidence of side-effects were the secondary outcomes investigated for 72 h after surgery. Hospital stay after surgery was also recorded. Any syndrome of local anesthetic systemic toxicity (LAST) including seizures, cardiovascular collapse, metallic taste, and tinnitus was recorded either immediately following TAP block implementation or after operation. Intravenous metoclopramide (10 mg) was available as needed for PONV treatment. In addition, postoperative comfort and satisfaction scale (0 = pretty dissatisfied, 3 = highly satisfactory) was documented through assessment of pain, relevant side effects, gastrointestinal motility and movement limitation for post-surgical 72 h.

### Statistical analyzes

A pilot study was conducted and a power analysis was implemented to calculate the sample size. The mean pain scores at rest of the four treatment groups (Group S, Group 0.375% R, Group 0.4% CL, and Group 0.6% CL) at post-surgical 24 h were 3.0, 2.9, 2.6, and 2.1, respectively. We determined a difference of at least 30% (error standard deviation = 1.2) for pain scores among groups. An *a priori* algorithm was used to estimate the required sample size for one-way analysis of variance (ANOVA). A sample size of 34 patients per group was found to be sufficient to detect a significant difference (*α* = 5%) with a statistical power (*β*-value) of 0.8. Presuming a 20% failure rate, we considered increasing the sample size to 41 patients per group.

The Shapiro–Wilk test was used to determine the normality of distribution of the data, and parametric statistics were applied. Homogeneity of variance was verified by the Levene test. Data from the NRS scores was analyzed by two-way repeated measures ANOVA with Bonferroni *post hoc* comparisons. Data from total dose of first postoperative sufentanil titration, sufentanil consumption by PCA, time until passage of flatus and urinary catheter removal were analyzed by one-way ANOVA with Bonferroni *post hoc* comparisons. Other quantitative data, such as age, weight, and mean concentration of sevoflurane and extubation time were also analyzed using one-way ANOVA with Bonferroni *post hoc* comparisons. Simultaneously, the *χ*^2^ test and Fisher’s exact test were used to analyze categorical variables, such as atropine administration and the incidence of PONV. Data were expressed as the mean (SD) or the number of patients/percentage. A *p* value <0.05 was considered statistically significant. SPSS 21.0 software (SPSS, Inc., Chicago, IL, United States) was used for all statistical analysis.

## Results

### Patient characteristics

**Figure 1:**
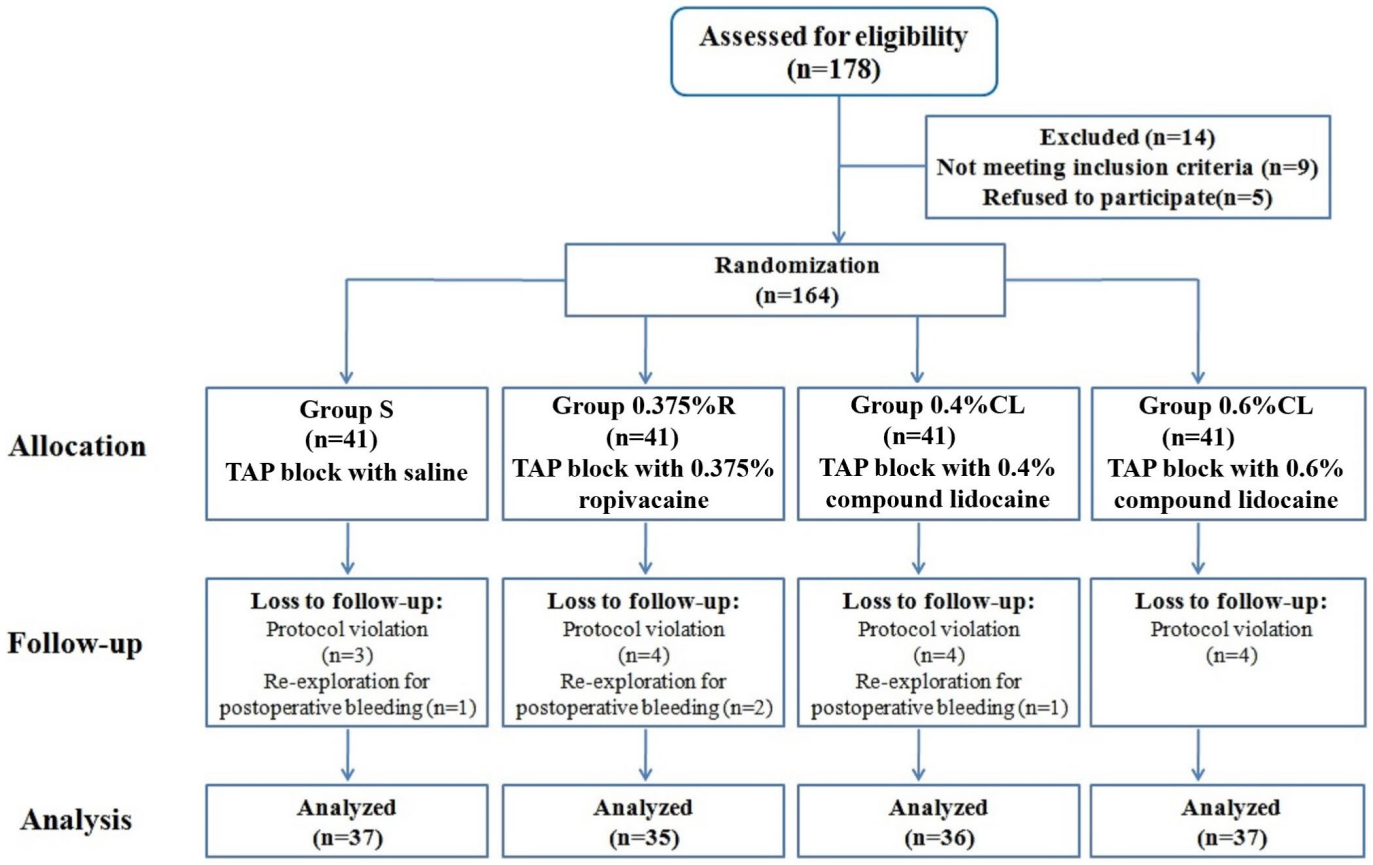
Consolidated standards of reporting trials (CONSORT) flow diagram.

Among the 178 patients recruited, 164 patients were eligible for inclusion. Nineteen patients were withdrawn after protocol violation or re-detection for postoperative bleeding, and 145 patients completed the study (Figure 1). The four groups were balanced in terms of patient characteristics (Table 1). There were no unexpected adverse safety cases of LAST in this study.

**Table 1:** Patient characteristics and anesthetic data.

Characteristic	Group S (*n* = 37)	Group 0.375% R (*n* = 35)	Group 0.4% CL (*n* = 36)	Group 0.6% CL (*n* = 37)	*P* value
Age (yr)	46 (11)	47 (10)	47 (9)	48 (9)	0.784
Height (cm)	161 (5)	160 (5)	161 (4)	162 (4)	0.199
Weight (kg)	61 (6)	60 (8)	58 (8)	62 (7)	0.194
ASA status (II)	25/68	27/77	28/78	27/73	0.740
Disease pathology					
Ovarian cancer	12/32	10/29	14/39	15/41	0.688
Fallopian cancer	6/16	6/17	5/14	5/14	0.967
Endometrial cancer	16/43	17/49	16/44	15/41	0.922
Other/mixed cancer	3/8	2/5	1/3	2/5	0.802
Duration of surgery (min)	210 (20)	203 (19)	211 (18)	213 (17)	0.144
Mean dose of remifentanil infusion (ug kg^−1^ min^−1^)	0.11 (0.04)	0.09 (0.02)^*^	0.09 (0.03)^*^	0.08 (0.03)^**^	0.002
Mean concentration of sevoflurane (%)	2.8 (0.5)	2.6 (0.4)	2.7 (0.4)	2.6 (0.4)	0.312
Intraoperative administration					
Phenylephrine	5/14	5/14	4/11	6/16	0.968
Atropine	2/5	3/9	4/11	2/5	0.756
Post-anesthesia recovery time					
Eye opening (min)	7.2 (2.2)	6.8 (2.7)	6.5 (2.5)	6.3 (2.7)	0.489
Extubation (min)	8.8 (2.8)	7.7 (2.5)	7.7 (2.2)	7.5 (2.8)	0.131

Group S: TAP block with saline; Group 0.375% R: TAP block with 0.375% ropivacaine; Group 0.4% CL: TAP block with 0.4% compound lidocaine; Group 0.6% CL: TAP block with 0.6% compound lidocaine. A bilateral TAP block was performed before the induction of anesthesia. All patients underwent elective gynecological laparotomy under sevoflurane anesthesia. Values are presented as the mean (SD), or the number of patients/%. *p < 0.05 vs. Group S, **p < 0.01 vs. Group S. ASA, American Society of Anesthesiologists; TAP, transversus abdominis plane.

### Intraoperative and post-anesthesia clinical variables

No significant differences was detected between groups with respect to duration of surgery, mean volume of sevoflurane, intraoperative MAP and HR, the proportion of patients administered phenylephrine and atropine during operation, post-anesthesia eye opening time and extubation time (Table 1; Supplementary Figure S1). As compared to Group S, total remifentanil use (Table 1) was less in Group 0.375% R (*p* = 0.024), Group 0.4% CL (*p* = 0.016), and Group 0.6% CL (*p* = 0.002). However, total remifentanil consumption did not differ between Groups 0.375% R, 0.4% CL, and 0.6% CL.

### Postoperative pain intensity

**Figure 2:**
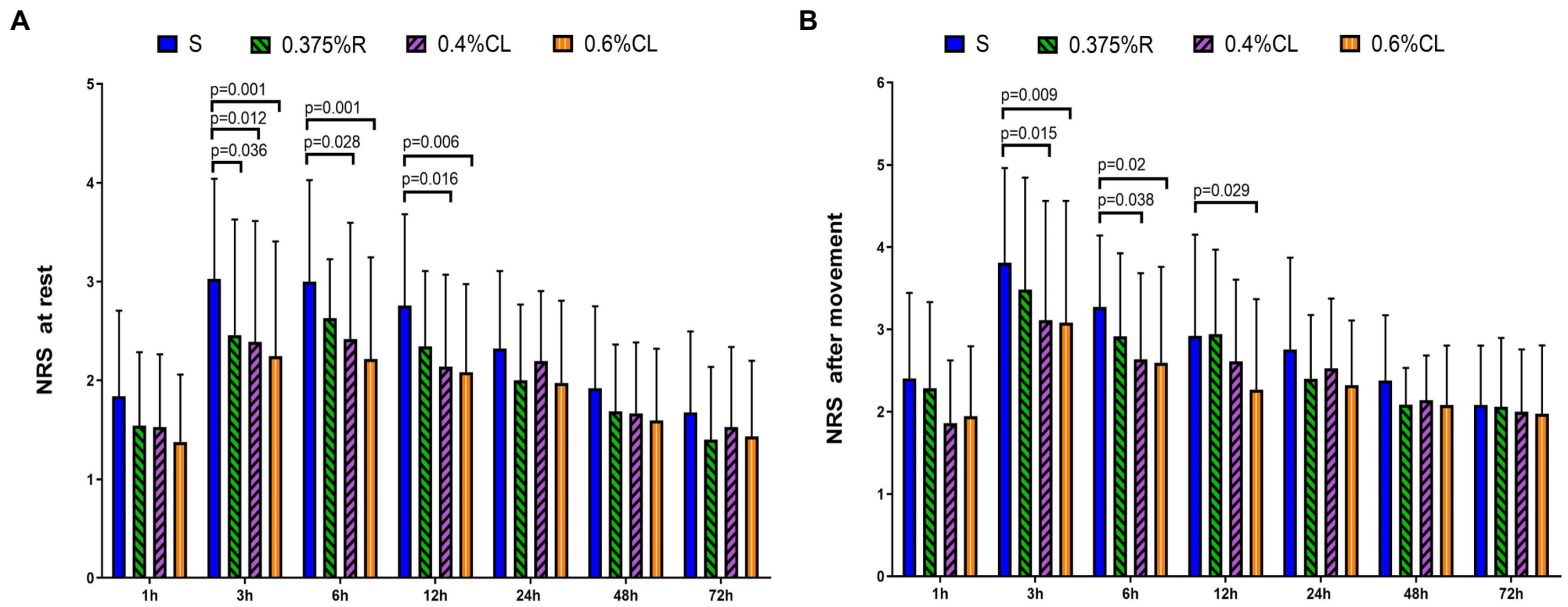
Postoperative pain intensity. The numerical rating scale (NRS) score for pain at rest **(A)** and after movement **(B)** was documented at 1, 3, 6, 12, 24, 48, and 72 h after surgery. Group S: TAP block with saline; Group 0.375% R: TAP block with 0.375% ropivacaine; Group 0.4% CL: TAP block with 0.4% compound lidocaine; Group 0.6% CL: TAP block with 0.6% compound lidocaine. A bilateral TAP block was performed before the induction of anesthesia. All patients underwent elective gynecological laparotomy under sevoflurane anesthesia. Values are presented as mean (SD). TAP, transversus abdominis plane.

As shown in Figure 2, NRS scores at rest were lower in Group 0.375% R than Group S at 3 h (*p* = 0.036) after surgery. Furthermore, as compared to Group S, patients in Group 0.4% CL exhibited the lower NRS scores at rest at postoperative 3 h (*p* = 0.012), 6 h (*p* = 0.028), and 12 h (*p* = 0.016) and NRS scores after movement at 3 h (*p* = 0.015) and 6 h (*p* = 0.038) after surgery. Strikingly, NRS scores at rest were lower in Group 0.6% CL than Group S at 3 h (*p* = 0.001), 6 h (*p* = 0.001), and 12 h (*p* = 0.006) after surgery. NRS scores after movement were also lower in Group 0.6% CL than Group S at 3 h (*p* = 0.009), 6 h (*p* = 0.02), and 12 h (*p* = 0.029) after surgery.

### Postoperative sufentanil consumption

Table 2 displays postoperative analgesics administration. As compared to Group S, the demand of sufentanil titration in the PACU in Group 0.375% R (*p* = 0.042), Group 0.4% CL (*p* = 0.007), and Group 0.6% CL (*p* = 0.002) was significantly decreased, whereas there was no significant difference between Groups 0.375% R, 0.4% CL, and 0.6% CL. More importantly, when compared with Group S, sufentanil requirement by PCA was lower in Group 0.375% R during the first 6 h after PCA, in Group 0.4% CL during the first 24 h after PCA and in Group 0.6% CL during the first 48 h after PCA.

**Table 2:** Postoperative sufentanil consumption.

Method of sufentanil application	Group S (*n* = 37)	Group 0.375% R (*n* = 35)	Group 0.4% CL (*n* = 36)	Group 0.6% CL (*n* = 37)	*p*-value						
					*P*_1_	*P*_2_	*P*_3_	*P*_4_	*P*_5_	*P*_6_	*P*_0_
By titration in the PACU (μg)											
	4.1 (2.5)	2.1 (2.1)	1.8 (1.9)	1.5 (1.7)	0.042	0.007	0.002	>0.99	>0.99	>0.99	<0.001
By PCA (μg)											
0–6 h	12.3 (2.6)	8.8 (2.5)	8.5 (2.4)	7.7 (1.3)	<0.001	<0.001	<0.001	>0.99	0.261	0.677	<0.001
6–12 h	8.0 (2.8)	6.4 (2.8)	4.2 (2.5)	3.6 (2.1)	0.061	<0.001	<0.001	0.002	<0.001	>0.99	<0.001
12–24 h	12.6 (4.5)	10.9 (4.0)	9.4 (3.7)	7.3 (4.0)	0.49	0.006	<0.001	0.682	0.002	0.204	<0.001
24–48 h	12.8 (5.0)	11.5 (4.4)	10.4 (5.1)	5.7 (2.6)	>0.99	0.149	<0.001	>0.99	<0.001	<0.001	<0.001
48–72 h	8.0 (2.9)	7.7 (3.2)	7.2 (2.2)	6.4 (2.8)	>0.99	>0.99	0.068	>0.99	0.249	0.589	0.062

Postoperative pain was controlled by sufentanil titration in the PACU, followed by sufentanil infusion *via* PCA. Group S: TAP block with saline; Group 0.375% R: TAP block with 0.375% ropivacaine; Group 0.4% CL: TAP block with 0.4% compound lidocaine; Group 0.6% CL: TAP block with 0.6% compound lidocaine. A bilateral TAP block was performed before the induction of anesthesia. All patients underwent elective gynecological laparotomy under sevoflurane anesthesia. Values are presented as the mean (SD). *P*_1_, Group S vs. Group 0.375% R; *P*_2_, Group S vs. Group 0.4% CL; *P*_3_, Group S vs. Group 0.6% CL; *P*_4_, Group 0.375% R vs. Group 0.4% CL; *P*_5_, Group 0.375% R vs. Group 0.6% CL; *P*_6_, Group 0.4% CL vs. Group 0.6% CL; *P*_0_, comparing groups. PACU, postanesthetic care unit; PCA, patient-controlled analgesia; TAP, transversus abdominis plane.

### Gastrointestinal motility and other surgical outcomes

As shown in Table 3, when compared with Group S, patients in Group 0.4% CL (*p* = 0.021) and Group 0.6% CL (*p* = 0.009) showed the earlier time to first flatus after surgery. Similarly, when compared with Group S, patients in Group 0.6% CL experienced the shorter length of post-surgical stay in hospital (*p* = 0.034). However, no significant differences of time until mobilization and urinary catheter removal were detected between four groups.

**Table 3:** Postoperative *surgical outcomes*.

	Group S (*n* = 37)	Group 0.375% R (*n* = 35)	Group 0.4% CL (*n* = 36)	Group 0.6% CL (*n* = 37)	*P*-value
Mean time until passage of flatus (h)	29.2 (7.2)	28.3 (7.6)	24.5 (6.1)^*^	24.1 (5.9)^**^	0.002
Mean time to mobilization (h)	38.8 (10.6)	38.1 (9.9)	35.4 (8.6)	34.1 (9.4)	0.122
Mean time until urinary catheter removal (h)	40.5 (10.5)	40.4 (8.9)	37.8 (7.7)	36.0 (8.8)	0.104
Length of postoperative stay in hospital (d)	6.9 (1.5)	6.7 (1.7)	6.6 (1.4)	5.9 (1.2)^*^	0.029
Postoperative comfort and satisfaction score	1.9 (0.6)	2.0 (0.6)	2.1 (0.5)	2.5 (0.6)^***^	<0.001

Gastrointestinal motility and other surgical outcomes were evaluated after surgery. Group S: TAP block with saline; Group 0.375% R: TAP block with 0.375% ropivacaine; Group 0.4% CL: TAP block with 0.4% compound lidocaine; Group 0.6% CL: TAP block with 0.6% compound lidocaine. A bilateral TAP block was performed before the induction of anesthesia. All patients underwent elective gynecological laparotomy under sevoflurane anesthesia. Values are presented as the mean (SD). *P*, comparing groups. *P_1_ < 0.05 vs. Group S; **P_1_ < 0.01 vs. Group S; ***P_1_ < 0.001 vs. Group S. TAP, transversus abdominis plane.

### Postoperative comfort and satisfaction

The incidence of postoperative nausea in Group S were higher than that in Group 0.6% CL (*p* = 0.009), whereas no differences were found between Groups S, 0.375% R and 0.4% CL (Table 4). The incidence in other postoperative side-effects did not differ among the four groups (*p* > 0.05; Table 4). Additionally, with respect to comfort and satisfaction score (Table 3), patients reported greater satisfaction during the first postoperative 72 h in Group 0.6% CL than in Group S (*p* < 0.001), Group 0.375% R (*p* = 0.002), or Group 0.4% CL (*p* = 0.046).

**Table 4:** Postoperative side-effects.

Side-effect	Group S (*n* = 37)	Group 0.375% R (*n* = 35)	Group 0.4% CL (*n* = 36)	Group 0.6% CL (*n* = 37)	*P*-value
Hypotension	4/11	2/6	3/8	2/5	0.800
Bradycardia	2/5	1/3	2/6	3/8	0.813
Nausea	12/32	6/17	5/14	3/8^*^	0.043
Vomiting	5/14	2/6	2/6	1/3	0.294
Ileus	5/14	3/9	1/3	2/5	0.342
Shivering	1/3	2/6	3/8	2/5	0.774
Headache	2/5	1/3	0/0	1/3	0.575
Somnolence	3/8	0/0	3/8	1/3	0.265
Dizziness	2/5	2/6	1/3	3/8	0.802
Respiratory depression	2/5	1/3	0/0	0/0	0.298

The incidence of the main adverse effects was evaluated during the first 72 h after surgery. Group S: TAP block with saline; Group 0.375% R: TAP block with 0.375% ropivacaine; Group 0.4% CL: TAP block with 0.4% compound lidocaine; Group 0.6% CL: TAP block with 0.6% compound lidocaine. A bilateral TAP block was performed before the induction of anesthesia. All patients underwent elective gynecological laparotomy under sevoflurane anesthesia. Values are presented as the number of patients/%. *P*, comparing groups. ^*^P_1_ < 0.05 vs. Group S. TAP, transversus abdominis plane.

## Discussion

The primary findings of this present study are: First, an US-guided bilateral TAP block with 0.375% ropivacaine alleviates postoperative pain for 3 h and reduces opioids requirement for 6 h in patients following gynecological laparotomy. Second, pretreatment of TAP block with 0.4% compound lidocaine inhibits post-surgical pain for 12 h and decreases opioids usage for the first 24 h after surgery. Third, pretreatment with of TAP block with 0.6% compound lidocaine provides excellent post-surgical analgesia for 12 h and reduces opioids consumption for the first 48 h after surgery. Fourth, preoperative therapy of TAP block with 0.6% compound lidocaine accelerates the return of bowel function, shortens hospital stay after surgery, and reduces the incidence of postoperative nausea, which may be as an essential element of multimodal analgesia therapy for patients who would undergo open abdominal surgery in ERPs setting.

Although laparoscopic surgery has the advantages of minimal invasive incision, fewer complications, faster recovery, and less pain after operation, exploratory laparotomy was preferred for gynecological cancer due to the complexity of surgical procedures including hysterectomy, bilateral salpingectomy or salpingo-oophorectomy, lymph node dissection, omentectomy, and other tumor debulking (Huepenbecker et al., 2019). Major incision and intra-abdominal procedures may cause moderate and severe somatic and visceral pain, and increase opioid prescription (Brummett et al., 2017; Mattson et al., 2021). When opioid is administered in excess, patients frequently experience several dose-limiting adverse-effects including nausea, ileus, respiratory depression, addiction, tolerance and hyperalgesia, which delaying bowel function recovery and hospital discharge (Colvin et al., 2019; Kent et al., 2019). Multimodal opioid-sparing analgesia approaches involve using multiple, simultaneous agents and procedures to target pain transmission signaling peripherally and centrally to improve analgesia and reduce opioid demand to minimize risks of side-effects (Beverly et al., 2017; Wu et al., 2019; Allen et al., 2020; Mattson et al., 2021). However, recent investigations have revealed that multimodal analgesia can be particularly challenging in several patients due to the efficacy of anticonvulsant agents and concerns regarding the safety of acetaminophen and NSAIDs (Bindu et al., 2020; Ishitsuka et al., 2020; Maheshwari et al., 2020). Another facet of multimodal analgesia is neuraxial block and regional anesthesia (Mattson et al., 2021). Although perioperative epidural anesthesia exhibits potent analgesic properties for patients with surgeries in gynecological oncology, the prevalence of postoperative hypotension and urinary retention is unexpectedly increased (Huepenbecker et al., 2019). TAP block has been advocated in postoperative pain control after intra-abdominal operations (Oksar et al., 2016; Edney et al., 2019), but single-shot TAP block may be insufficient to provide a durable block (Ghisi et al., 2016; Hain et al., 2018). Whether TAP block is beneficial to postoperative analgesia after gynecological laparotomy needs further investigation.

Given that US-guided block can elevate the accuracy of local anesthetic injection into the TAP and reduce the risks of LAST (Ghisi et al., 2016; Xu et al., 2020), we performed bilateral TAP block under US imaging guidance. In the present study, TAP block was conducted prior to surgery since it may produce better identification of tissue planes without post-surgical tissue oedema, and include more analgesic data on whether TAP block rescues intraoperative opioid use. Ropivacaine but not bupivacaine was selected for TAP block due to its lower lipophilicity, less motor blockade, and less relevance to LAST (Golembiewski and Dasta, 2015). We chose the concentration of 0.375% as ropivacaine injection (equivalent to total 150 mg) for bilateral TAP block according to clinical practice and previous reports (Sun et al., 2017; Xu et al., 2020). Perioperative therapy of acetaminophen and flurbiprofen axetil along with opioids was used for analgesia throughout the present study, consistent with multimodal analgesia protocols and ERPs guidelines (Beverly et al., 2017; Edney et al., 2019; Wu et al., 2019; Allen et al., 2020). Not surprisingly, we observed that preoperative TAP block with 0.375% ropivacaine reduced intraoperative opioid administration while ensuring sufficient anti-nociception and minimizing hemodynamic fluctuations during surgery. TAP block with 0.375% ropivacaine also exhibited the transient pain-relief and mild opioid-sparing effects for the first post-surgical 3–6 h. However, it did not have any positive effect on postoperative gastrointestinal function return, urinary catheter removal, early ambulation and hospital stay, which might be attributed to short duration of action of ropivacaine without significant reduction in cumulative opioid consumption. Although the higher dose of ropivacaine may acquire superior benefits, the plasma concentration of ropivacaine will exceed the widely quoted toxic concentration of 2.2 μg mL^−1^ if more than 2.5–3 mg kg^−1^ of ropivacaine is used in TAP block, which increasing the risk of LAST (Griffiths et al., 2013). Collectively, TAP block occupies a clear advantage in major laparotomy and is worth popularizing if duration of block is effectively prolonged.

Liposomal bupivacaine is a long-acting multivesicular liposome formulation that provides prolonged bupivacaine release (Manna et al., 2019). Recently, TAP block with liposomal bupivacaine as an element of multimodal analgesia down-regulated total opioid consumption during 72 h after cesarean delivery (Nedeljkovic et al., 2020). However, the benefit should be considered in the context of extreme high costs of liposomal bupivacaine. Notably, compound lidocaine hydrochloride injection has been utilized for local wound infiltration with highly efficient performance and relatively competitive price (Wang, 2015, 2020; Dai et al., 2019). Herein, our current study, for the first time, reported that preoperative TAP block with both 0.4 and 0.6% compound lidocaine attenuated postoperative pain, as characterized by the similar decrease in pain scores for 12 h after surgery. Moreover, patients undergoing TAP block with 0.4 and 0.6% compound lidocaine experienced the decreased opioid medications for 24 h and 48 h after surgery, respectively, which this reduction in opioid use did not sacrifice adequate postoperative pain control. These detailed results also demonstrated that the post-surgical analgesic and opioid-sparing benefits of compound lidocaine is significantly superior than that of ropivacaine. The longer duration of analgesia may be explained by the existence of menthol and glycerin in compound lidocaine injection. Several literatures have identified the local anesthetic and analgesic effects of menthol, and the activity of menthol to enhance tissue permeability and facilitate local anesthetic delivery (Gaudioso et al., 2012; Liu et al., 2013; Castro and Dent, 2017; Oz et al., 2017; Pergolizzi Jr. et al., 2018; Wright et al., 2018). The viscosity of glycerin allows local anesthetics to remain locally for a longer time. Unfortunately, our results cannot explain these observations, which requires to be further investigated. Intriguingly, although TAP block using 0.4 and 0.6% compound lidocaine provided similar enhanced-recovery of gastrointestinal function, patients receiving 0.6% compound lidocaine experienced the shorter hospital stay and the greater comfort and satisfaction. Perhaps, it is because 0.6% compound lidocaine exhibited the lower incidence of postoperative nausea and the less opioid usage. Previously, the highest serum concentration of lidocaine recorded after TAP block with 400 mg lidocaine was 5.5 μg mL^−1^, which is just above the toxic concentration of 5.0 μg mL^−1^, but no any case of LAST was found in that study (Kato et al., 2009). The recommendation for the maximum dose of lidocaine is 300 mg, although this recommendation is neither evidence-based nor takes the site of injection into account (Rosenberg et al., 2004). Oksar and his colleagues also demonstrated the safety of TAP block with 200 mg lidocaine for patients undergoing laparoscopic cholecystectomy (Oksar et al., 2016). We chose the concentration of 0.4 and 0.6% as compound lidocaine injection (equivalent to 80 mg and 120 mg lidocaine in 20 mL each side, respectively) for bilateral TAP block based on our clinical practice. Of note, we did not detect any syndrome of LAST either after TAP block or after operation in our study, suggesting the safety of compound lidocaine at total dose of 160 and 240 mg for TAP block.

There are several limitations to consider in this study. One is the failure to measure the serum concentration of lidocaine after TAP block using compound lidocaine injection, which should be addressed by future prospective studies. It is identified that post-surgical opioid-reducing benefit can translate into less opioid prescriptions upon discharge and that discharge prescriptions can be tailored on account of postoperative inpatient opioid consumption (Hill et al., 2018; Kent et al., 2019). We did not assess opioid prescriptions upon discharge since these patients commonly receive standard discharge prescriptions, although these questions warrant further investigations. We did not measure the length of midline incision, which may affect postoperative pain. Moreover, further trials are needed to ascertain whether these positive results are generalizable to other abdominal surgical populations. Another possible weakness is that all experiments were conducted at a single hospital and might not be representative for postoperative practices at other institutions. Additionally, the recent systematic review recapitulates that many adjuncts (such as alpha-2 agonists, dexamethasone, and ketamine) to local anesthetic infiltration for post-surgical analgesia can prolong and enhance the activity of local anesthetics (Bai et al., 2020). In future initiatives, we will evaluate whether these adjuncts can further prolong the duration of compound lidocaine for TAP block in clinical situations, all of which will assist in our efforts to decrease opioid abuse without sacrificing pain control.

In conclusion, the current findings demonstrate that preoperative therapy of TAP block using 0.6% compound lidocaine attenuates postoperative pain, reduces perioperative opioid usage and the incidence of postoperative nausea, accelerates bowel function recovery, and shortens length of postoperative stay in hospital in patients with gynecological laparotomy. Consequently, TAP block with compound lidocaine may be implemented as a promising therapeutic candidate for multimodal analgesia strategy in routine clinical practice.

## Data availability statement

The raw data supporting the conclusions of this article will be made available by the authors, without undue reservation.

## Ethics statement

The studies involving human participants were reviewed and approved by Tianjin Medical University General Hospital Ethic Committee. The patients/participants provided their written informed consent to participate in this study.

## Author contributions

LZ and GW conceived the experiment. ZJ, YZ, JL, and YW collected the data. LZ, QL, TG, and YY analyzed the data. LZ, ZJ, and GW wrote the paper. All authors contributed to the article and approved the submitted version.

## Funding

This work was supported by research grants from the National Natural Science Foundation of China (82171205, 81801107, 81371245, 82071243, and 81571077) and the Tianjin Natural Science Foundation Project (Tianjin, China; 19JCYBJC25500 and 20JCYBJC00370).

## Conflict of interest

The authors declare that the research was conducted in the absence of any commercial or financial relationships that could be construed as a potential conflict of interest.

## Publisher’s note

All claims expressed in this article are solely those of the authors and do not necessarily represent those of their affiliated organizations, or those of the publisher, the editors and the reviewers. Any product that may be evaluated in this article, or claim that may be made by its manufacturer, is not guaranteed or endorsed by the publisher.

## Supplementary material

The Supplementary material for this article can be found online at: https://www.frontiersin.org/articles/10.3389/fnmol.2023.967917/full#supplementary-material

## Footnotes

^1^ www.clinicaltrials.gov
