# Supplementary figures and images for: A randomized controlled trial evaluating the effects of transversus abdominis plane block with compound lidocaine hydrochloride injection on postoperative pain and opioid consumption and gastrointestinal motility in patients undergoing gynecological laparotomy
Source: Front Mol Neurosci. 2023 Jan 25;16:967917. doi: 10.3389/fnmol.2023.967917 (PMC9905139; doi:10.3389/fnmol.2023.967917)

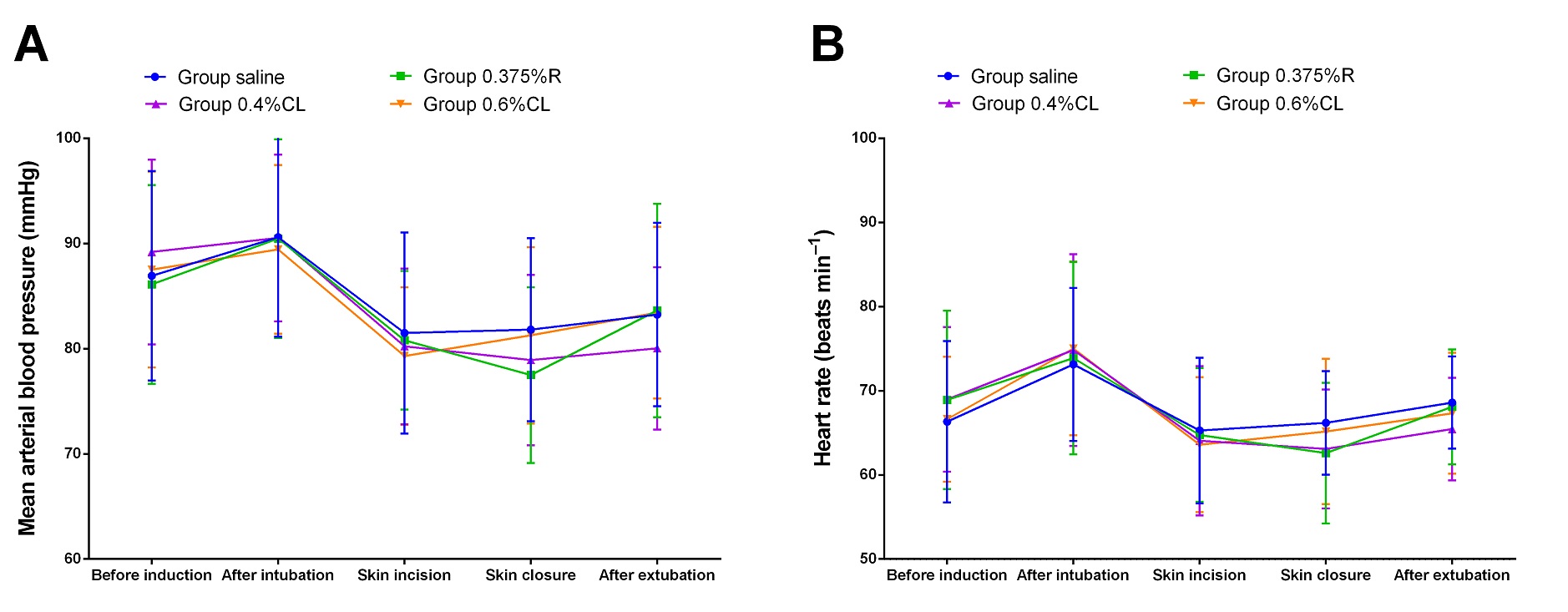

Supplement: Supplementary file 1 [file Image_1.JPEG]
